# Development of an optical system for the non‐invasive tracking of stem cell growth on microcarriers

**DOI:** 10.1002/bit.26328

**Published:** 2017-05-23

**Authors:** Akinlolu Oyekunle Oluseun Odeleye, Sara Castillo‐Avila, Mathew Boon, Haydn Martin, Karen Coopman

**Affiliations:** ^1^ Centre for Biological Engineering Loughborough University Loughborough LE11 3TU United Kingdom; ^2^ Institute of Biomedical Engineering University of Oxford Oxford United Kingdom; ^3^ University of Huddersfield Huddersfield United Kingdom

**Keywords:** epi‐illumination, mesenchymal stem cells, cell therapy, image analysis, microcarriers, cell culture

## Abstract

The emergence of medicinal indications for stem cell therapies has seen a need to develop the manufacturing capacity for adherent cells such as mesenchymal stem cells (MSCs). One such development is in the use of microcarriers, which facilitate enhanced cell densities for adherent stem cell cultures when compared with 2D culture platforms. Given the variety of stem cell expansion systems commercially available, novel methods of non‐invasive and automated monitoring of cell number, confluence, and aggregation, within disparate environments, will become imperative to process control, ensuring reliable and consistent performance. The in situ epi‐illumination of mouse embryonic fibroblasts and human mesenchymal stem cells attached to Cytodex 1 and 3 microcarriers was achieved using a bespoke microscope. Robust image processing techniques were developed to provide quantitative measurements of confluence, aggregate recognition, and cell number, without the need for fluorescent labeling or cell detachment. Large datasets of cells counted on individual microcarriers were statistically analyzed and compared with NucleoCounter measurements, with an average difference of less than 7% observed from days 0 to 6 of a 12‐day culture noted, prior to the onset of aggregation. The developed image acquisition system and post‐processing methodologies were successfully applied to dynamically moving colonized microcarriers. The proposed system offers a novel method of cell identification at the individual level, to consistently and accurately assess viable cell number, confluence, and cell distribution, while also minimizing the variability inherent in the current invasive means by which cells adhered to microcarriers are analyzed. Biotechnol. Bioeng. 2017;114: 2032–2042. © 2017 The Authors. *Biotechnology and Bioengineering* Published by Wiley Periodicals, Inc.

## Introduction

The last decade has seen the cell therapy industry (CTI) emerge as a major component of the global healthcare market. Cell therapy is a platform technology, utilizing living cells for therapeutic applications. These can range from permanent cell therapies such as the replacement of limbal cells for damaged corneas, or transient cell therapies, that is, adult stem cells used to modulate the immune system. Due to their immunosuppressive, multipotency, and growth capacity, mesenchymal stem cells (MSCs) have emerged as an important cell type for therapeutic applications (Heathman et al., 2016; Jackson et al., 2007; Pittenger et al., 1999). With an annual turnover of over a billion dollars per year and an estimated market in the United States alone in excess of 100 million patients, the growth of the CTI is set to continue (Mason et al., 2011). As a burgeoning sector of global healthcare, the infrastructure required to fulfill its potential: such as scalable manufacturing, cost models, and regulatory procedures, are still in development (Mason et al., 2011; Panchalingam et al., 2015).

Traditionally, adherent cells are grown on 2D T‐flask surfaces; however, individual treatments require billions and even trillions of cells for therapeutic effectiveness. As such, alternative manufacturing methods are required to ensure the economic and practical feasibility of stem cell therapy (Brandenberger et al., 2011). The commercial success of adherent cell therapies is strongly dependent upon achieving these high harvest cell numbers, particularly for allogeneic treatments (Rowley et al., 2012). To meet this manufacturing challenge, a number of strategies for the scale‐up of stem cell manufacturing within closed and controllable systems have emerged. Three‐dimensional (3D) structures such fibrous scaffolds, microcarriers and stacked plates are being increasingly used to help increase available adherent surface area and thus facilitate enhanced scale‐up of adherent cell therapies (Simaria et al., 2014). Microcarrier‐based cell culture systems are particularly pertinent as not only do they facilitate higher cell concentrations, they can also be integrated into existing bioprocess manufacturing systems such as stirred bioreactors and spinner flasks (Rafiq et al., 2013). The enhanced surface area to volume ratio engendered enables higher cell densities and reduces the production footprint, when compared with traditional planar culture systems (Rowley et al., 2012). Scalable manufacturing processes such as those using stirred tanks, coupled with an appropriate storage strategy, also benefit from the economies of scale observed in typical biopharmaceutical processes (Heathman et al., 2016).

The principles of process analytical technology (PAT) and quality by design (QbD), are an important requirement for biopharmaceutical production (Teixeira et al., 2009). This ensures that quality is built into the design by defining the interdependency of critical parameters, with the aid of statistical methods to determine the parameter space in which quality is maintained (Glassey et al., 2011; Mandenius, 2016; Mandenius et al., 2009). Within the biologics industry, the real‐time measurement of viable cell concentration is one of a number of key parameters that facilitate the development of monitoring and control approaches (Kell et al., 1990). The development of in situ microscopy methods for the on‐line measurement of suspension cell culture concentrations, has been noted in the literature (Camisard et al., 2002; Guez et al., 2004; Joeris et al., 2002; Wiedemann et al., 2009). When dealing with a therapeutic substance whereby the cells themselves are the product, monitoring cellular health, viability, morphology, and where possible function, are necessary to optimize the design space. Comparable detection methods are thus necessary for the growing use of microcarrier‐based cell cultures. As with suspension cell cultures, tracking viable cell growth is pertinent within adherent cell culture, in addition to detecting cell and microcarrier aggregation as this impacts nutrient transfer to the cells (Kirouac and Zandstra, 2008). However, ascertaining cell density during a microcarrier‐based cell culture is challenging, due to the necessity to sample from the culture and then either detach cells from the carrier or label the cells with fluorescent dye to enable cell counting. These additional cell handling steps, significantly increase the sources of error attributable to the final cell number (Justice et al., 2011) and, being an off‐line measurement, do not enable the setup of automated feedback loops to control feeding, medium exchange, or microcarrier addition into the culture system based on cell number. The study presented delineates the development of an optical system for the non‐invasive monitoring of mesenchymal stem cells grown on microcarriers. A camera system incorporating epi‐illumination microscopy technology has been manufactured for cell imaging, while image acquisition protocols and post‐processing algorithms have been developed to enable robust and automated cell detection and analysis.

## Materials and Methods

### Monolayer Cell Preparation

Bone‐marrow derived human mesenchymal stem cells (hMSCs) were obtained from Lonza (Lonza, Cologne AG), acquired from a healthy donor with informed consent provided by the patient. These cells have been approved by the local Ethical Committee for research use. The NIH/3T3 mouse embryonic fibroblasts (MEFs) were obtained from ATCC (American Type Culture Collection, Manassas, VA). Both of these cells were expanded in monolayer culture using Nunc™ Thermo Scientific™ T175 plastic T‐flasks, with cell seeding at 5,000 cells/cm^2^. Dulbecco's Modified Eagle's Medium (DMEM) modified to contain 4 mM of l‐glutamine, 4.5 g/L glucose and 3.7 g/L of sodium bicarbonate (Sigma–Aldrich, Gillingham, UK) and supplemented with 10% v/v newborn calf serum (Sigma–Aldrich), was prepared for the MEF growth media. While the hMSCs were cultured using DMEM containing 1 g/L of glucose, no l‐Glutamine (Lonza, Slough, UK) and supplemented with 10% v/v fetal bovine serum (Lonza) and 2 mM UltraGlutamine (Lonza). Initial expansion of both cell‐lines were prepared in 35 mL of their respective DMEM media recipes in T175 (175 cm^2^ area) T‐flasks. The cells were incubated at 37°C in a humidified incubator in which CO_2_ was controlled at 5%. On day 3 of the culture, a complete medium change was performed and the cells were subsequently passaged on day 6 of the culture (the point at which cells become confluent). The passage process involved washing the cells (hMSCs or MEFs) with 15 mL of Ca^2+^ and Mg^2+^ free phosphate buffer saline (Lonza) twice, before incubating the cells with 15 mL of 0.25 % Trypsin/EDTA for 10 min, to aid in cell detachment from the T‐flask surface. A total of 15 mL of fresh growth medium was added to the trypsin‐cell solution to quench the enzyme. The cell suspension was centrifuged at 250*g* for 5 min at room temperature. The supernatant was aspirated and discarded before resuspending the cell pellet with 5 mL of fresh growth medium. The viable cell count was performed using a NucleoCounter® NC‐3000™ in which Acridine Orange and DAPI (4′,6‐diamidino‐2‐phenylindole) were used to stain the entire cell population and non‐viable cell population, respectively.

### Microcarrier Spinner Flask Preparation

The T‐flask expanded cells (as prepared in the previous section) were used to inoculate spinner flasks using three different types of microcarriers: Cytodex 1 (GE Healthcare, Buckinghamshire, UK), Hillex II (Pall SoloHill, Ann Arbor, MI) and Plastic Plus (Pall SoloHill) microcarriers in 100 mL spinner flasks (BellCoVineland, NJ) (tank diameter of *T* = 60 mm) with a magnetic stirrer bar and vertical paddle (diameter of *D* = 50 mm). The glass BellCo spinner flask was siliconized with Sigmacote (Sigma), left overnight to dry, rinsed with de‐ionized water, and sterilized via autoclaving. Microcarriers representing an attachment area of 500 cm^2^ were prepared according the manufacturers’ specification. The microcarriers were conditioned with 50 mL of growth media for 24 h in an incubator with the impeller speed set to 30 rpm (the minimum impeller rate at which microcarriers suspension occurs). The 50 mL of conditioning media was replaced with 96 mL of growth media. Then, the T‐flask expanded cells were used to inoculate the 100 mL spinner flask, seeding at 6,000 cells/cm^2^. The spinner flask culture was then incubated at 37°C, 5% CO_2_, and 30 rpm. A 50% media exchange was performed on day 3 of the culture, and every 2 days thereafter. NucleoCounter cell counts, phase contrast, and epi‐illumination microscopy imaging were performed every 2 days from day 2 of the 12‐day culture. The counting of cells attached to the microcarriers involved first lysing the cells by adding 200 μL of Reagent A100 (Chemometec, Allerød, Denmark) to 200 μL of the microcarrier cell culture. The sample was vortex mixed for 30 s and left to rest for 30 s. A total of 200 μL of the stabilizing buffer Reagent B (Chemometec) was then added to the lysed cells, vortex mixed for 30 s, and left to rest for 30 s. The released nuclei were then aspirated into the NucleoCounter Via1‐Cassette™, which contains DAPI to stain the nuclei for counting. The longer the cells are exposed to Reagent A100, the more nuclei are released. However, nuclei are not stable in Reagent A100 and will decline over time. The microcarrier cell expansion and subsequent cell counting protocols described, have been used and validated within our laboratory against viable cell measurements of enzyme detached cells, using trypan blue exclusion (Heathman et al., 2016; Nienow et al., 2014; Rafiq et al., 2013).

### In Situ Epi‐Illumination Microscopy Set‐Up

**Figure 1:**
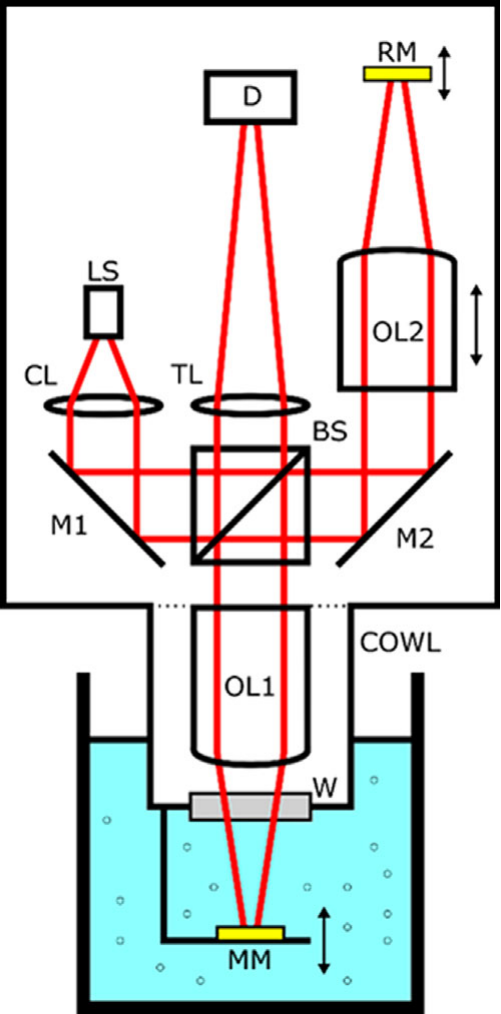
Schematic design of the bespoke in situ epi‐illumination microscope. The test arm is immersed in the tank where the microcarrier culture is suspended.

A custom‐built interference microscope was used to acquire the static microcarrier image acquisitions and a schematic representation is shown in Figure 1. In this apparatus, a fiber‐coupled superluminescent diode (SLED) light source (LS), having a central wavelength of 820 nm, linewidth of 25 nm, and an optical power of 2 mW is collimated by a lens (CL) and then split by a beam splitter (BS) to form the two arms of a Linnik configuration interferometer, having matched Mitutoyo ×10 magnification long working distance microscope objectives in each arm (OL1, OL2). This configuration allows for an extended long working distance, and a simpler submersible front‐end, compared to other interferometer configurations as there are no optics required to be placed in front of the objective lens. The measurement arm comprises a sapphire window (W), which allows light to exit the submersible cowl and a mirror (MM) which enables the measurement of transparent samples using epi‐illumination. The reference arm comprises a mirror (RM) which is placed at the focal point of the microscope objective (OL2). In addition, there is a path length adjustment, via a zoom barrel which allows the axial movement of the reference objective (OL2) in order to bring the system into interference; this is necessary because the coherence length of the source is only a few tens of microns. The reference and measurement beams recombine at the beam splitter, after retro‐reflection from their respective mirrors, and the resulting interferogram is cast upon a CMOS area detector (D) having 1,280 × 1,024 pixels by a tube lens (TL). In Figure 1, double headed arrows denote where critical axial displacement adjustment was engineered, namely for the reference (RM) and measurement (MM) mirrors and the reference objective lens (OL2). Two folding mirrors (M1, M2) are also present in order to enable a more compact final layout for the apparatus. Optical performance of the system was assessed using a USAF 1951 standard test target. Lateral resolutions down to approximately 1.6 μm in air and 2.4 μm in water were achieved, the latter case providing a field of 598 × 479 μm^2^. The apparatus is held vertically and immersed into the bioreactor tank; the microcarriers flow between the sapphire window and the measurement mirror. The interference microscope can be also operated as an in situ epi‐illumination microscope by simply blocking the reference arm beam. In this way, it was possible to assess the possible benefits of using interference or epi‐illumination alone to detect feature contrast.

Epi‐illumination imaging of colonized microcarriers were conducted in a 300 mL beaker (FisherBrand, Leicestershire, UK). The camera cowl was positioned in the empty beaker before being filled with enough PBS to submerge the cowl window. A cell culture sample of 5 mL was aseptically transferred from an active microcarrier spinner flask and added to the beaker for imaging. Although the system is capable of generating interference‐based imaging, for cell counting, images appeared clearer using the epi‐illumination microscopy system alone. In order to acquire images of moving microcarriers, a LUMICS LU0808M250 (250 mW, central wavelength of 808 nm, and linewidth of 0.5 nm FWHM) pulsed diode laser replaced the SLED used for static image acquisition. The laser was configured to produce a pulse duration of 400 ns at a repetition rate of 5 Hz. The culture beaker containing the PBS and culture sample was placed onto a GyroStir D HOT (SciQuip) digital hotplate magnetic stirrer. The beaker contents were then set to stir at 100 rpm using a magnetic stirrer bar (30 × 6 mm^2^). Once set, all laser protection coverings were put in place, and the camera set to acquire images for 400 s yielding a total of 2,000 images.

## Results and Discussion

### Image Acquisition—Microcarrier Screening

**Figure 2:**
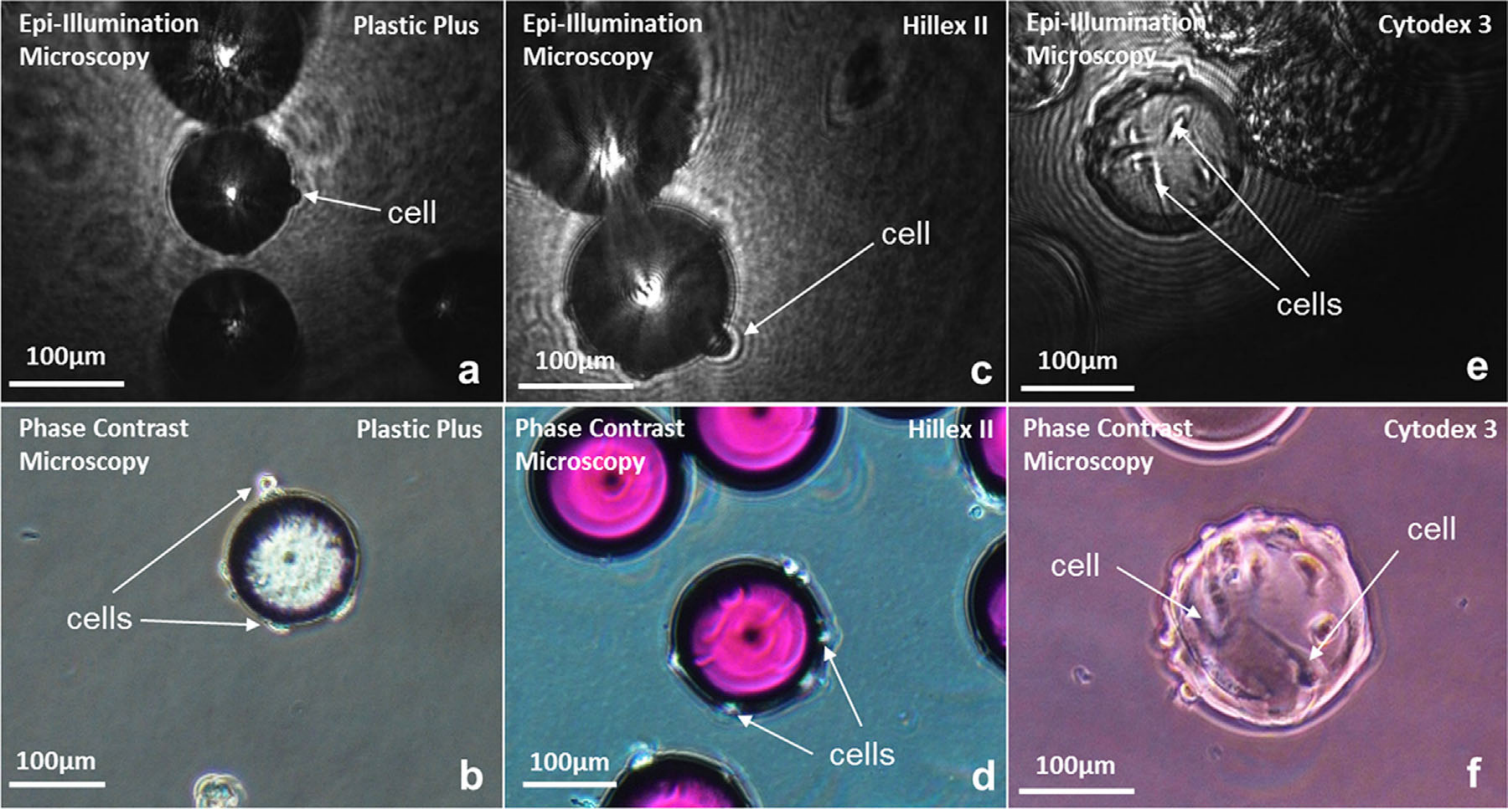
Epi‐illumination microscopy and phase contrast image acquisitions of 3T3 mouse embryonic fibroblasts attached to Plastic Plus microcarriers (**a** and **b**), Hillex II (**c** and **d**), and Cytodex 3 (**e** and **f**), respectively.

In order to assess the capacity of the system to image adherent cells, epi‐illumination imaging of colonized microcarriers were initially conducted with MEFs, grown on Plastic Plus, Hillex II, and Cytodex 3 microcarriers. The microcarriers were imaged under static conditions, with colonized microcarriers resting on the surface of a mirror (Fig. 1) designed to enhance illumination of the cells. Figure 2 shows phase contrast and epi‐illumination microscopy of MEFs attached to Plastic Plus, Hillex II, and Cytodex 3 microcarriers. It is clear from both techniques, the ability to observe the cells attached to Plastic Plus microcarriers (Fig. 2a and b) is limited to the perimeter of the microcarrier image. This is primarily due to the opacity of the plastic microcarriers, reducing the degree of cell illumination and/or reflection.

The phase contrast images present in Figure 2d illustrate the greater transparency exhibited by the Hillex II microcarriers: in these images, as with those in Figure 2a and b, cells are observable on the microcarrier perimeter. On the Hillex II front surface, qualitative information regarding the presence of cells on the microcarrier can be gained, if not the ability to detect individual cells. This additional information does not translate to the epi‐illumination microscopy derived images, with cells primarily observable on the perimeter of the microcarrier image. In both the Plastic and Hillex II epi‐illumination image acquisitions, the optimum particle position was at the interference plane. This is the position at which the interference patterns occur when both beams are aligned, located approximately at the microcarrier equatorial plane.

With regard to the MEFs attached to Cytodex 3 microcarriers, as with the studies conducted by Nikolai et al. (1991) and Rudolph et al. (2008), the transparent nature of Cytodex provides greater clarity for distinguishing cells (when compared with more opaque microcarriers, e.g., plastic and polystyrene). However, the 3D structure of the microcarrier surface still precludes the accurate detection of all cells attached on the entirety of the microcarrier.

Figure 2f highlights the difficulty in differentiating cells from the background, microcarrier and each other, due to the coloration of the cells at different locations on the microcarrier. This is also evident in the work of Rudolph et al. (2008), a study in which an in situ phase contrast microscope was used to image Cytodex 1 microcarriers colonized with MSCs. The study noted the grayscale values of cells and their borders differed depending on lighting, reflection and shadows: thus inhibiting their ability to detect individual cells. In the aforementioned study, a correlation between the grayscale histogram of isolated (cropped) microcarrier images and their “Level of Coverage” (i.e., confluence) was determined to monitor cell growth. In the study presented, the focal point of the light source was positioned at or above the microcarrier equatorial plane: thus facilitating the enhanced differentiation of individual cells, through the illumination of cells on the upper hemisphere of the microcarrier.

At the lens position used, cells are distinguishable from both the background and microcarriers, based upon their relatively high grayscale pixel value. In order to determine the optimum camera exposure time, epi‐illumination microscopy images of MEFs attached to Cytodex 3 microcarriers at camera exposure times increasing from 0.01 to 0.12 ms were acquired. Exposure times in the range of 0.01–0.03 ms, equating to a total illumination energy of 20–90 nW, were found to give the optimum contrast of cells‐to‐background. This is important to note in the context of acquiring images of moving colonized microcarriers, so that the pulse duration of the illumination method can be optimally specified.

### Quantitative Confluence Measurement

The confluence of an adherent cell culture is typically performed through qualitative visual inspection. Quantitatively measuring cell confluence directly on microcarriers would facilitate enhanced control of surface area availability to the cells, negating the need for sampling, cell detachment, and counting. Thus, a MATLAB algorithm was developed (initially from 2D T‐flask images of MEFs and MSCs) to assess the ability of epi‐illumination microscopy to distinguish cell confluence. The protocol takes advantage of the low variation (standard deviation) in grayscale pixel values present in the image background. A high‐pass filter was applied to analyze kernels 15 × 15 pixels in size and retain regions of relatively high standard deviation. Segmentation techniques have been developed previously to assess confluence on phase contrast images of adherent cells (Bradhurst et al., 2008; Jaccard et al., 2014; Topman et al., 2011). Bradhurst et al. (2008) noted limitations in distinguishing the background in near confluent images, while Jaccard et al. (2014) described a reduction in measurement accuracy at low confluence levels due to the small intricate structures. Studies have utilized empirically derived threshold values that produce optimal results and remain constant thereafter (Jaccard et al., 2015; Topman et al., 2011). To account for widely disparate cell confluence and image quality, the filter threshold was determined specifically from the image under analysis: in this case the value was set to 35% of the standard deviation of grayscale pixel values from the entire image (σ_image_). The analysis kernel was convolved sequentially across the whole image with a 93% overlap in both the *x* and *y* direction. The confluence is then simply calculated as the percentage of pixels classified as being cells and not background. For additional accuracy, Jaccard et al. (2014) take the segmentation analysis further by removing the bright halos associated with phase contrast images of stem cells. However, halos are not present in the epi‐illumination microscopy images generated, so do not require this correction.

**Figure 3:**
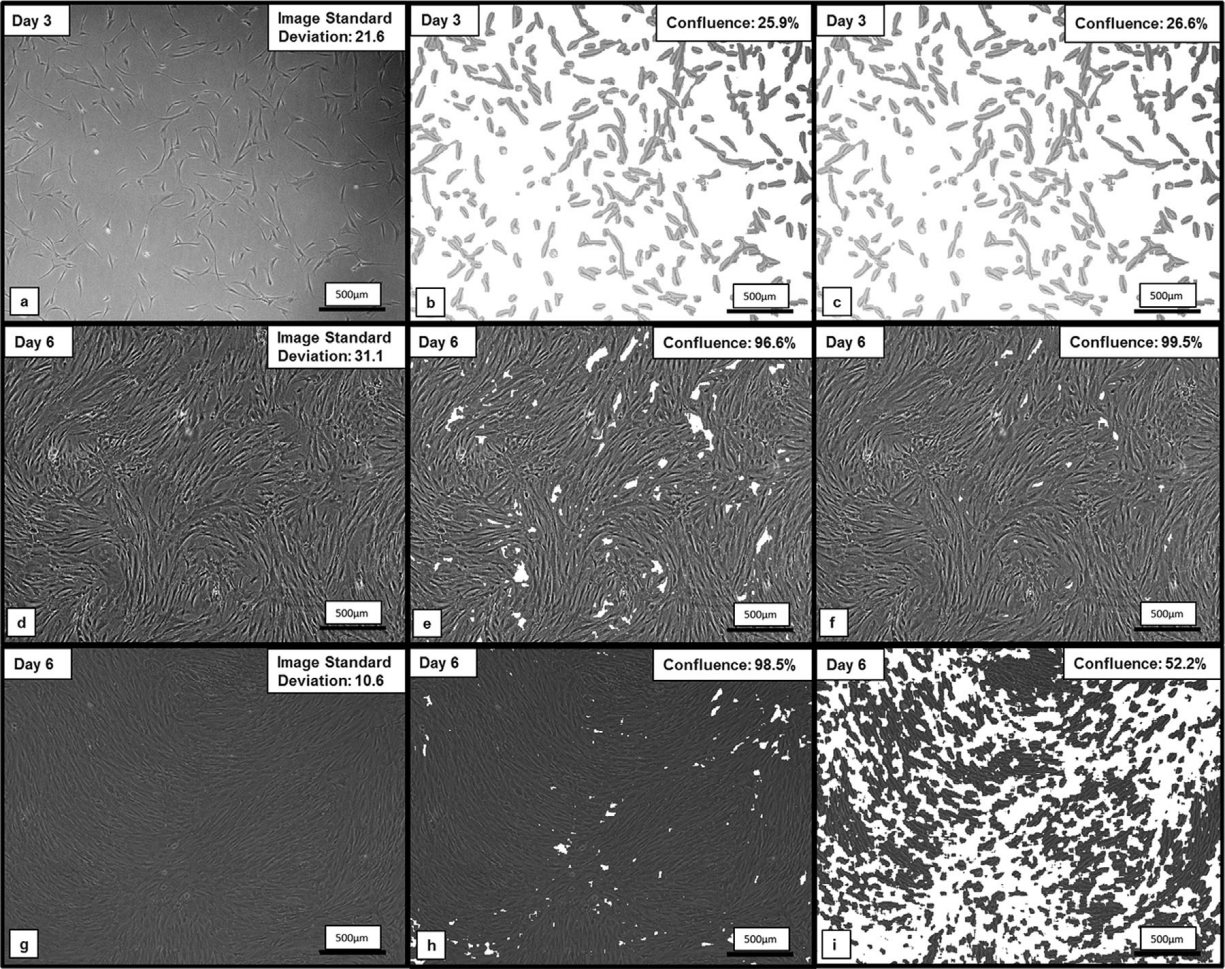
Output images of the confluence algorithm, used to discriminate days 3 and 6 MSCs attached to a T175 flask, from the background. (**a**, **d**, and **g**) Represent the original images; (**b**, **e**, and **h**) are the output using a high‐pass filter threshold of 0.4 × *σ*_image_; and (**c**, **f**, and **i**) are the output using a constant high‐pass filter threshold of 0.4 × 21.1.

**Figure 4:**
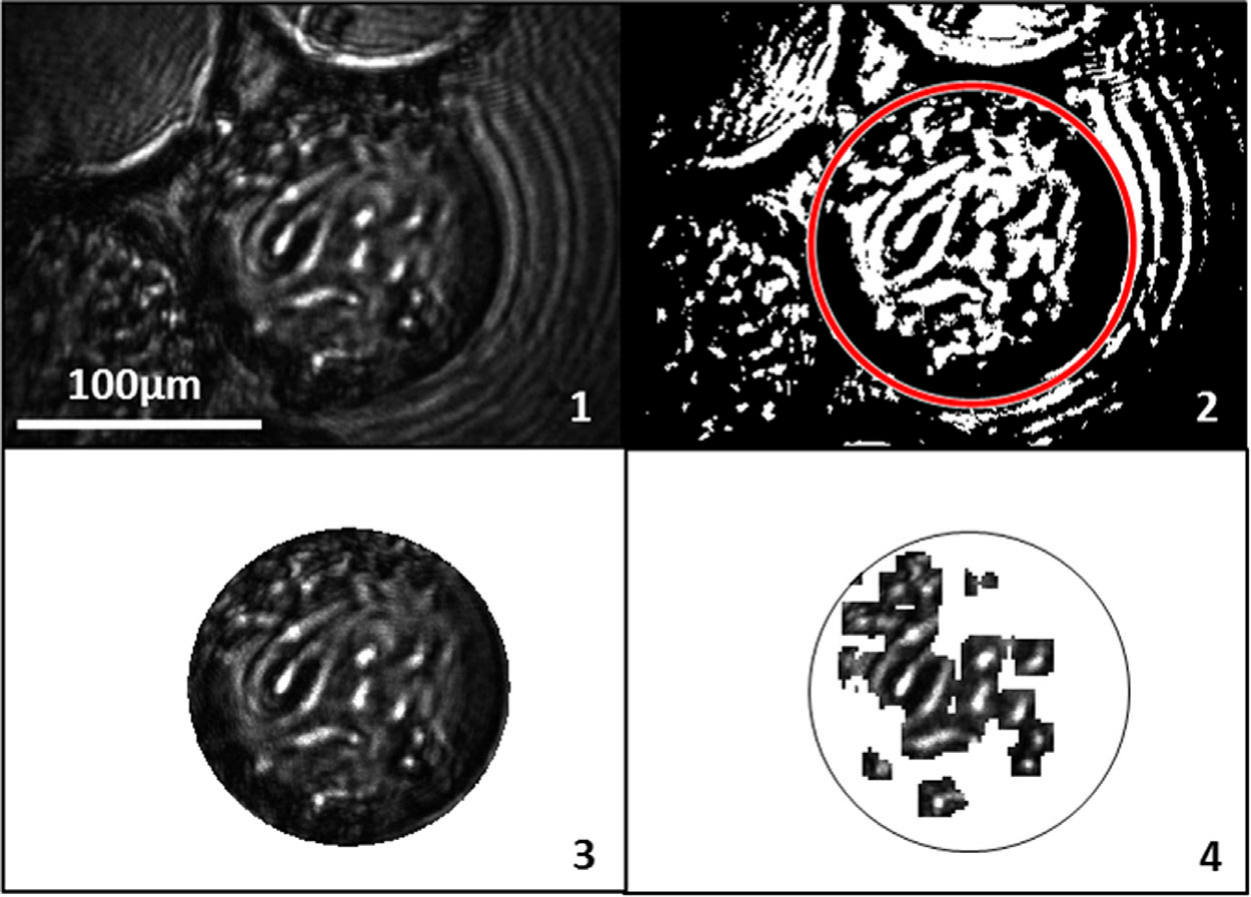
Sequential image processing steps for confluence measurement of 3T3 mouse embryonic fibroblasts attached to Cytodex 3 microcarriers.

Figure 3 illustrates 2D T175 flask images of MSCs, as well as the confluence algorithm output images, at 3 and 6 days, post‐cell seeding: Figure 3a, d, and g is the original image. Figure 3b, e, and h represents the output using a high‐pass filter threshold of 0.4 × *σ*_image_. Figure 3c, f, and i shows the output using a constant high‐pass filter threshold of 0.4 × 21.1 (21.1 is the average *σ*_image_ of the three original images shown in Fig. 3a, d, and g). Utilizing a constant high‐pass filter threshold, as noted by Bradhurst et al. (2008), results in difficulty when discerning the background at near full confluence (Fig. 3e). An additional 2.9% of “background” is detected when using the variable threshold criteria. Furthermore, relatively dark confluent images appear to pose a problem for the non‐variable threshold method, with confluence measurements of 98.5% and 52.2% determined, using the variable and non‐variable threshold approaches, respectively. This illustrates the need to for a variable threshold criterion, particularly for high confluence images and images of varying quality. The development of a quantitative assessment of cell confluence removes the inherent subjectivity associated with subjective qualitative methods. To analyze the colonized microcarriers, the Hough transform was utilized to isolate the microcarrier imaged, before applying the confluence measurement algorithm described. These steps are illustrated in Figure 4.

### Image Analysis—Cell Count

**Figure 5:**
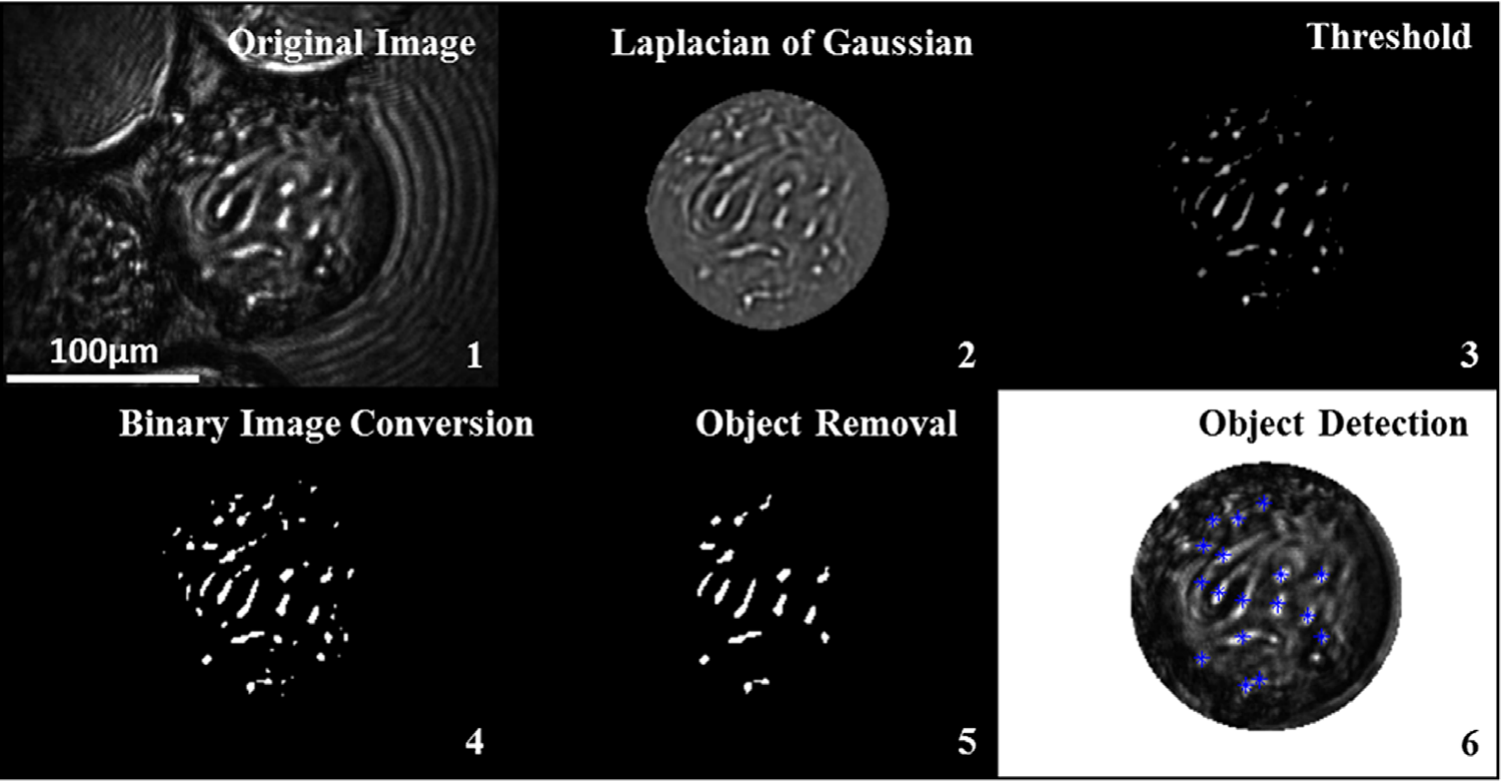
Image analysis sequence for cell detection algorithm. Cells detected are indicated with stars.

The in situ epi‐illumination microscope engenders the generation of large image datasets that provide real‐time information in relation to microcarrier cell adherence. In order to analyze these data, a robust process of microcarrier identification, isolation, and subsequent analysis was required. The first stage is microcarrier isolation using the circle detection method delineated in the previous section, before cropping the surrounding area from the microcarrier. Cells were distinguishable by their relatively high grayscale pixel values. In order to successfully identify the cells, a Laplacian of Gaussian (LoG) filter was applied. Figure 5 shows the image processing sequence used to detect and count MEFs attached to a Cytodex 3 microcarrier.

The LoG is a derivative filter first introduced by Marr and Hildreth (1980). It is used to detect areas of rapid change (i.e., edges of objects) through the Laplacian component, while the Gaussian component reduces background noise to minimize erroneous object detection (Woolford et al., 2007). This method for detecting the cells was implemented to attenuate the effect of disparate image quality resulting from variation in light intensity and the positioning of the focal point relative to the cells. Both the kernel size and sigma values were tuned by assessing the statistical impact of their variation, visual assessment of cells identified, and by comparing the counted cells to NucleoCounter quantified cell numbers. A high‐pass (grayscale pixel value) filter was performed following the LoG operation, to further differentiate the cells, before additional refinement using a binary image conversion and object thresholding by pixel area.

As with the confluence measurement algorithm, the cell counting procedure was developed and tested extensively on cells attached to 2D T‐flask surfaces and on multiple spinner flask microcarrier cultures ranging from 1 to 12 days post‐seeding. On initial testing of the cell counting algorithm, the NucleoCounter and LoG algorithm derived hMSC counts from three separate T‐flasks resulted in, on average, a 2.2% higher LoG‐based cell count. In this scenario, a slightly higher cell count when using the algorithm is expected, due to its non‐invasive nature.

With respect to the analysis of colonized microcarriers, once counted, the cell number was multiplied by 2, as it was assumed that only cells on the upper hemisphere were counted. This method was then used to track cell growth throughout a 12‐day microcarrier spinner flask culture. To assess the reproducibility of the algorithm across different cell cultures, a separate repeat spinner flask culture was conducted and the developed algorithm applied, to count hMSCs attached to Cytodex microcarriers. The difference between cell counts derived from epi‐illumination and NucleoCounter methods, for runs 1 and 2 of hMSCs seeded onto Cytodex microcarriers (for 2 days), was 3% and 0%, respectively.

### Tracking Cell Growth

**Figure 6:**
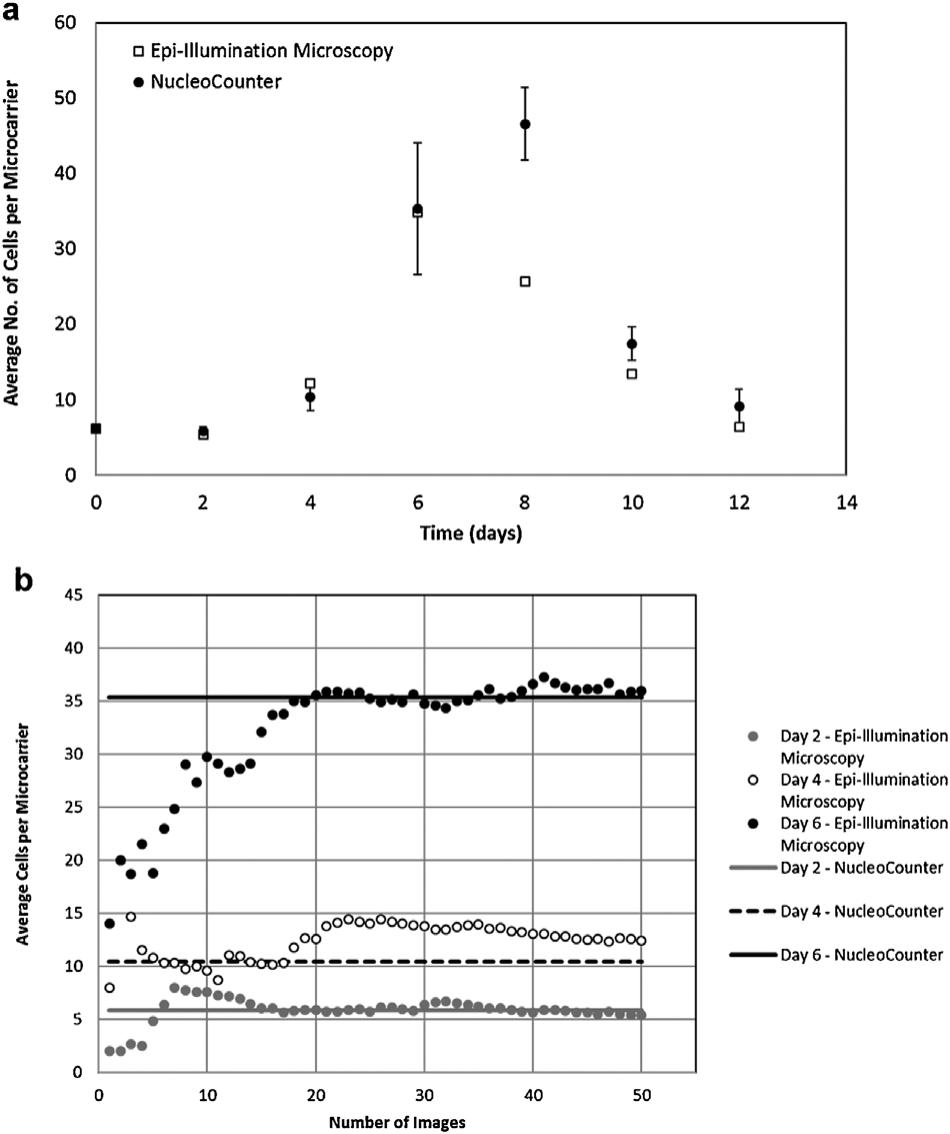
(**a**) Twelve‐day cell growth profile of MSCs attached to Cytodex 1 microcarriers (error bars represent the standard error of the triplicate NucleoCounter measurements) and (**b**) Profile of average cell number per microcarrier in relation to the number of microcarriers analyzed using in situ epi‐illumination microscopy on days 2, 4, and 6 of the aforementioned 12‐day culture.

Once a method of automated, mass image processing was established, 100 images were acquired to determine the number of images needed to obtain a statistically relevant cell count. A difference of 3% was noted between cell count measurements after 50 and 100 images. Figure 6b shows the average number of cells per microcarrier in relation to the number of microcarriers analyzed, during the expansion phase (days 2, 4, and 6) of a 12‐day spinner culture of MSCs grown on Cytodex 1 microcarriers. As is clear from Figure 6b, it takes between 20 and 40 individual microcarrier cell counts before the average cell number converges to a consistent value.

**Figure 7:**
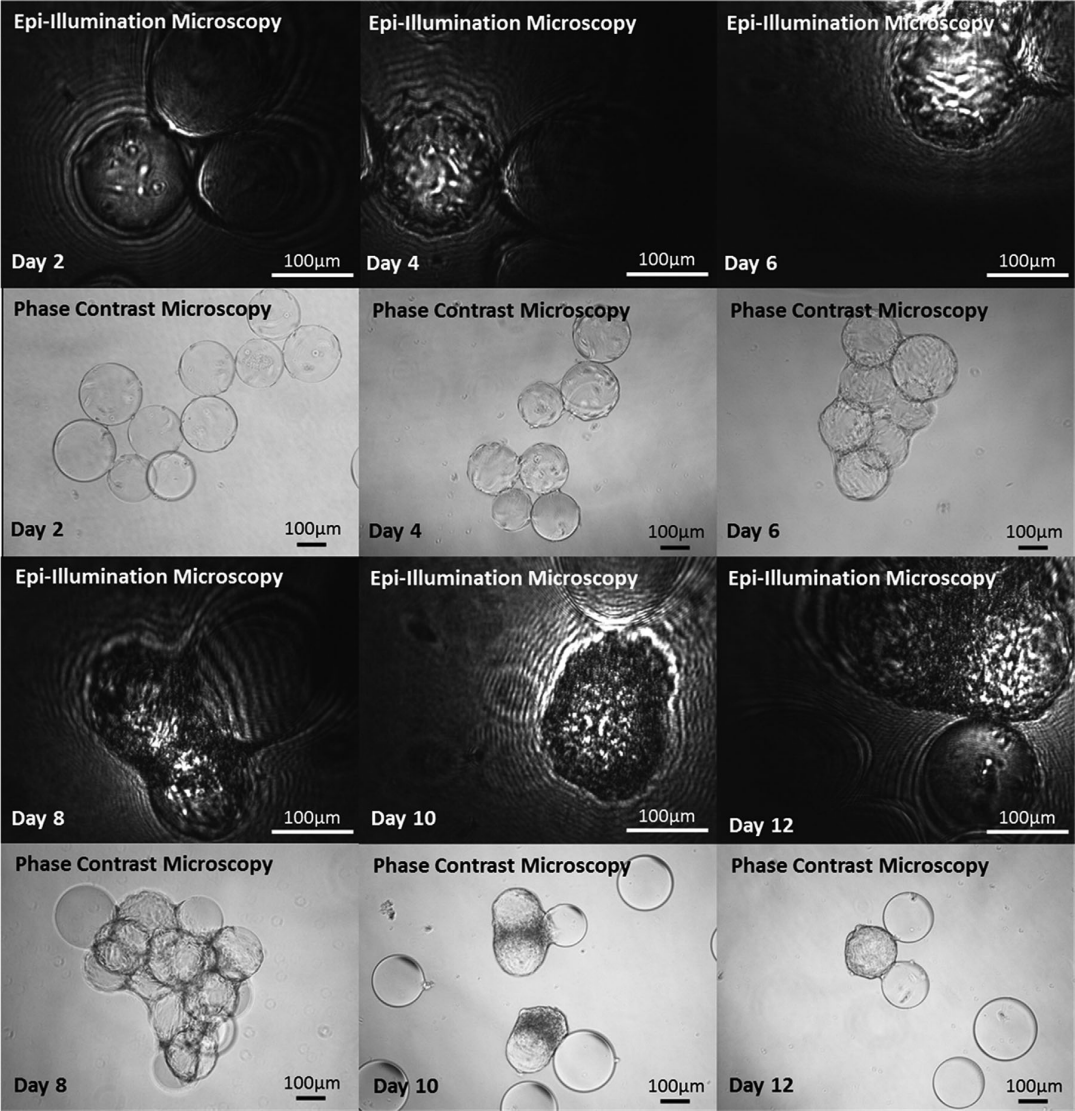
Epi‐illumination and phase contrast microscopy images of Cytodex 1 microcarriers colonized by MSCs during a 12‐day 100 mL spinner flask culture.

The 12‐day spinner flask culture was conducted in order to ascertain the effectiveness of the system and algorithms developed in tracking cell growth at varying degrees of confluence. Figure 7 illustrates epi‐illumination microscopy and phase contrast imaging of the colonized microcarriers in a 12 days MSC culture on Cytodex 1 microcarriers. It is noted that in this system, microcarrier aggregation commences on day 6 of the culture, with subsequent cell‐to‐cell aggregation and cell death visible on days 8, 10, and 12. With microcarrier aggregation, there is greater difficulty in discriminating individual cells, as well as imaging a statistically significant proportion of the microcarrier surface area. Figure 6a shows the viable cell growth profile as determined via microcarrier imaging and NucleoCounter measurements from culture samples. The imaging system and cell detection methods used compare well with NucleoCounter measurements during the lag phase and exponential growth phase of the culture (days 2–6). This shows that the assumption that the actual cell number is double that which can be detected using the algorithm holds true. However, once aggregation commences, the detectable cells per microcarrier become an underestimate of the NucleoCounter value. This is to be expected, given the degree of aggregation occurring during days 8–12 of the spinner flask culture. In the future, it is possible to envisage a control strategy set‐up which would automatically add additional microcarriers once aggregation or a threshold confluence level was achieved, to increase the amount of available surface area or to change the mixing regime to prevent further aggregation from taking place. Importantly, this in situ system is able to detect aggregation as further described later, information which is currently lost through off‐line sample processing and cell counting.

### Bead‐to‐Bead Transfer

**Figure 8:**
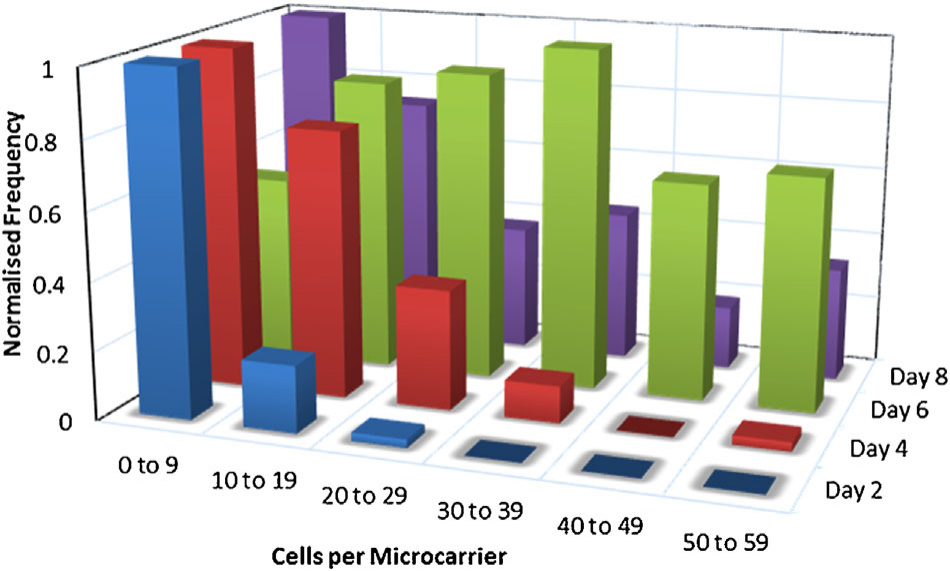
Normalized frequency of counted MSCs attached to Cytodex 1 microcarriers on days 2, 4, 6, and 8 of a 100 mL spinner flask culture.

Bead‐to‐bead transfer has, for some decades now, been utilized as the primary strategy for the scale‐up of cells grown on microcarriers (Schop et al., 2008; Wang and Ouyang, 1999). The primary bead‐to‐bead transfer mechanisms touted in the literature include the detachment of cells from the microcarrier; growth in media and subsequent reattachment; and migration of cells between microcarriers through direct contact (Frauenschuh et al., 2007). As a result, understanding the degree of microcarrier coverage and the distribution of cells upon the microcarriers can play an important role in optimizing the cell expansion phase. Figure 8 shows histograms (grouped by Day of Culture) of the frequency of microcarriers with the specified range of cells attached. The chart represents data from days 2 to 10 of the MSC culture, with the frequency normalized to the maximum value measured during the stated culture day. The histogram displays an increase in the proportion of microcarriers with more than 10 cells attached at day 4. This progresses further in day 6, which conveys the most even distribution of cells adhered to beads. As the cells take an initial period of attachment during the first 48 h of the culture, it required an additional 4 days for the newly colonized microcarriers to become confluent. This is in agreement with Wang and Ouyang (1999), who also note a 4‐day period for fresh microcarriers to become confluent.

### Aggregate Detection

**Figure 9:**
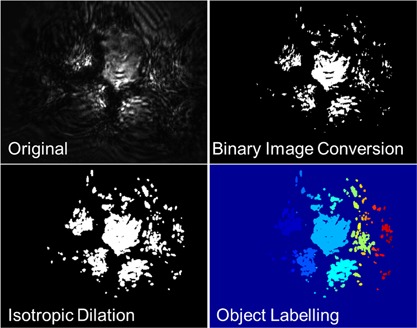
Image analysis sequence for aggregate detection algorithm.

In addition to the cell tracking and confluence measurements that the epi‐illumination microscope presented permits, the presence of aggregates can also be detected. Furthermore, an estimate of the population of microcarrier aggregates can also be quantified: adding a supplementary parameter from which surface area control measures can be derived. In order to ascertain the presence of aggregates, the following steps were incorporated into a MATLAB script to analyze the large image dataset acquired. First, a binary image conversion was applied to highlight the illuminated microcarriers. Object dilation further agglomerated the large bright regions of the spheres. Object labeling was then used to identify individual objects within the image. Microcarriers were identified by pixel area and eccentricity, while images containing aggregates were selected based on detection of more than two microcarriers within an image. The steps associated with this aggregate detection method are illustrated in Figure 9.

Using the aggregate recognition method described, the number of aggregates detected for every 50 images acquired, was attained: with values of 1.00, 1.79, 2.58, and 4.02 noted on days 2, 4, 6, and 8 of the cell culture, respectively. The aggregates detected using the algorithm matched exactly with human visual assessment of the images. This highlights a linear increase in aggregate detection frequency as the culture period progresses. Providing a potential threshold criterion with which control procedures would be initiated to increase the available surface area for cell attachment or augment fluid agitation to disperse the aggregates. The detection and quantification of aggregates within a cell culture is a step forward from other cell imaging technologies that rely on isolation of the culture sample prior to measurement, for example, Ovizio's 4D holographic microscopy system (iLine F) in which suspended cells are analyzed in a flow‐through system; a mechanism in which aggregates may be overlooked via breakage or due to their inability to be pumped during analysis.

### Pulsed Dynamic Acquisitions

**Figure 10:**
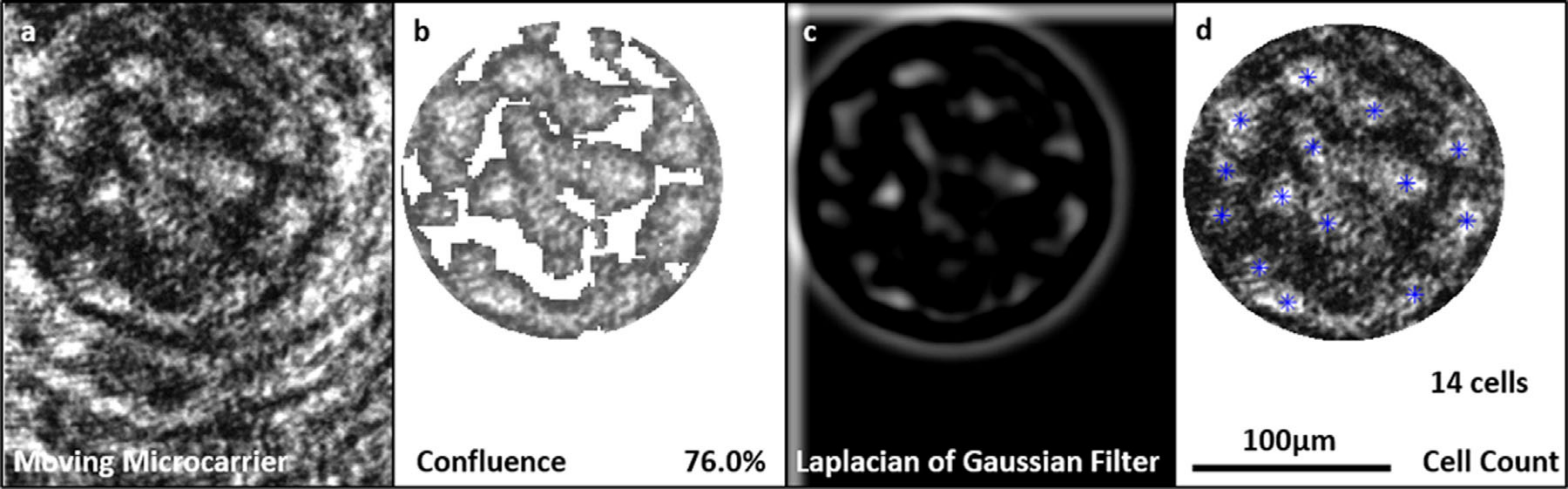
Epi‐illumination microscopy images of day 7 hMSCs attached to Cytodex 3 microcarriers moving in a magnetic bar stirred tank at 100 rpm. The figure shows (**a**) raw image of moving colonized microcarrier; (**b**) quantitative confluence measurement; (**c**) Laplacian of Gaussian filter for cell counting; and (**d**) counted cells on microcarrier.

The employment of an in situ epi‐illumination method for imaging and tracking stem cell growth on microcarriers has been described thus far for static microcarriers. Assessing the capacity of the in situ microscope to acquire images of moving microcarriers of necessary quality to be analyzed using the methods developed, would provide significant proof‐of‐concept for the potential use of such a system in future bioreactor technology. The pulsed laser used can provide 140 nJ of energy in a 400 ns pulse: this is over the minimum illumination requirements (20–90 nJ) outlined previously under static conditions. Figure 10a displays a raw image of hMSCs attached to a moving Cytodex 1 microcarrier using a pulsed laser diode operating with a 400 ns pulse width; Figure 10b an illustrative quantitative confluence measurement; Figure 10c the LoG filter; and Figure 10d the subsequent cell count. The difficulty in the system at present is imaging microcarriers that are in the optimal focal position; in the current system colonized microcarriers in the correct focal plane are acquired at a rate of approximately 1 in 1,000 images. Further work will address this by applying: physical methods, for example, fins and cowls to direct microcarrier flow more optimally across the focal plane; and image pre‐processing to pre‐select optimal images for subsequent cell analysis. Nonetheless, the analysis of moving colonized microcarriers without cell detachment or fluorescence demonstrates significant progression in adherent cell monitoring capabilities.

## Concluding Remarks

The study presented conveys the development of an in situ microscopy system combined with image processing methods for analyzing microcarriers colonized by mesenchymal stem cells. For the first time, a system was developed to non‐invasively and accurately quantify the cell number, microcarrier aggregation, and cell‐microcarrier migration characteristics, without the need for cell detachment or fluorescent labeling. The ability to monitor stem cells attached to microcarriers is greatly influenced by the physical properties of the chosen microcarrier. Its degree of transparency, geometric shape, and the cell location all impact upon the quality of images obtainable as well as the information that can be ascertained. The illumination of cells on the upper surface of the microcarrier via epi‐illumination microscopy, enabled the detection of individual cells through image processing techniques. While a proof‐of‐concept for the aforementioned analysis upon dynamically moving microcarriers was established, for this method of stem cell culture monitoring to be realized, the next stage is to apply the techniques developed to images acquired under agitated conditions for a continuous cell culture. This will be achieved through the optimized integration of a pulsed laser, synchronized to the image acquisition system, connected to a cell culture vessel in a continuous configuration. Applying the imaging technology to a bioreactor system for sterile monitoring of cells, along with further development of stem cell morphology analysis techniques would provide a novel and effective method for monitoring and controlling stem cell expansion and differentiation. Furthermore, the large datasets obtainable from such a system would align with the QbD strategies increasingly employed in the bioprocess industry.

This research was supported by the Engineering and Physical Sciences Research Council [grant numbers EP/L017555/1, EP/L017571/1].
